# Examination of the Novel Sigma-1 Receptor Antagonist, SI 1/28, for Antinociceptive and Anti-allodynic Efficacy against Multiple Types of Nociception with Fewer Liabilities of Use

**DOI:** 10.3390/ijms23020615

**Published:** 2022-01-06

**Authors:** Lisa L. Wilson, Shainnel O. Eans, Insitar Ramadan-Siraj, Maria N. Modica, Giuseppe Romeo, Sebastiano Intagliata, Jay P. McLaughlin

**Affiliations:** 1 Department of Pharmacodynamics, The University of Florida, Gainesville, FL 32610, USA; lisawilson@ufl.edu (L.L.W.); shaieans@cop.ufl.edu (S.O.E.); I.SirajRamadan@hotmail.com (I.R.-S.); 2 Department of Drug and Health Sciences, University of Catania, 95125 Catania, Italy; mmodica@unict.it (M.N.M.); gromeo@unict.it (G.R.)

**Keywords:** sigma, sigma-1 receptor, antagonist, allodynia, analgesia, neuropathic pain, sedation

## Abstract

Neuropathic pain is a significant problem with few effective treatments lacking adverse effects. The sigma-1 receptor (S1R) is a potential therapeutic target for neuropathic pain, as antagonists for this receptor effectively ameliorate pain in both preclinical and clinical studies. The current research examines the antinociceptive and anti-allodynic efficacy of SI 1/28, a recently reported benzylpiperazine derivative and analog of the S1R antagonist SI 1/13, that was 423-fold more selective for S1R over the sigma-2 receptor (S2R). In addition, possible liabilities of respiration, sedation, and drug reinforcement caused by SI 1/28 have been evaluated. Inflammatory and chemical nociception, chronic nerve constriction injury (CCI) induced mechanical allodynia, and adverse effects of sedation in a rotarod assay, conditioned place preference (CPP), and changes in breath rate and locomotor activity were assessed after i.p. administration of SI 1/28. Pretreatment with SI 1/28 produced dose-dependent antinociception in the formalin test, with an ED_50_ (and 95% C.I.) value of 13.2 (7.42–28.3) mg/kg, i.p. Likewise, SI 1/28 produced dose-dependent antinociception against visceral nociception and anti-allodynia against CCI-induced neuropathic pain. SI 1/28 demonstrated no impairment of locomotor activity, conditioned place preference, or respiratory depression. In summary, SI 1/28 proved efficacious in the treatment of acute inflammatory pain and chronic neuropathy without liabilities at therapeutic doses, supporting the development of S1R antagonists as therapeutics for chronic pain.

## 1. Introduction

Chronic neuropathic pain is a common cause for decreased quality of life and disability in the United States [1]. Chronic pain may be caused by disease or lesions within Aβ, Aδ and C fibers as well as central neurons found in the somatosensory system [2]. First and second line pharmacological treatments for this type of pain include antiepileptics and antidepressants, respectively [3,4], but these medications possess adverse effects including sedation, dizziness, and impaired locomotion [2], presenting a significant risk of falling and while operating machinery, thus lowering patient compliance. Similarly, third line treatment of neuropathic pain with opioids may produce adverse effects including tolerance, constipation, substance abuse and respiratory depression which can result in death [5,6]. Worse, the established treatments for chronic pain have been limited efficacy in approximately 50% of patients [4]. Overall, there is a clear need to find more effective analgesic therapeutic options with fewer liabilities to treat chronic pain [7].

Once thought to be a member of the opioid family [8], cloning of the sigma-1 receptor (S1R) in 1996 [9] established it as a ligand-regulated, intracellular chaperone protein expressed in primary sensory neurons involved in pain transduction [10]. S1R have been localized in the lumbar dorsal root ganglia in neuropathic states such as spared nerve injury and sciatica [10]. S1R in the peripheral and central nervous systems play a pivotal role in the hypersensitivity of pain and the function of nociception [11,12,13,14]. A growing number of studies indicate that inactivation or antagonism of S1R produces antinociception in both inflammatory-induced and neuropathic pain models [12,13,15,16]. However, many existing S1R antagonists currently used clinically, such as dextromethorphan, haloperidol, and imipramine, demonstrate poor S1R selectivity and significant off-target activity [12]. Recently, the push for highly selective S1R antagonists as therapeutics for neuropathic pain yielded MR309 (also known as E-52862), an S1R antagonist in phase IIa clinical trials for oxaliplatin-induced peripheral neuropathy [17], and [^18^F]FTC-146, which completed phase I clinical trials as a highly selective S1R PET imaging agent to visualize nerve damage in neuropathic states [18,19,20,21]. Extending this effort, we recently identified SI 1/13, a selective benzylpiperazine-based S1R antagonist with a S2R/S1R selectivity ratio of 886 that demonstrated efficacy against chronic constrictive nerve injury (CCI)-induced neuropathic pain and formalin-induced inflammatory pain without impairing locomotor activity [22]. In addition, SI 1/13 demonstrated significant cytotoxic effects towards DU145 and U87MG cancer cells, further corroborating its function profile as a S1R antagonist [23,24].

The current study sought to characterize an analog of SI 1/13, 1-(4-{[4-(hydroxymethyl)phenyl]methyl}piperazin-1-yl)-5-phenylpentan-1-one oxalate (named herein SI 1/28), for its ability to modulate chronic neuropathic and inflammatory pain states in mice. As reported previously (as compound 24; see [22]), radioligand binding studies found SI 1/28 to have high affinity for the S1R (*K*_i_ S1R = 6.1 nM) over the sigma-2 receptor (*K*_i_ S2R = 2,583 nM) for a S2R/S1R selectivity ratio of 423 deemed desirable for further examination. As in vitro functional assays to assess S1R activity remain under development [25], we relied on established in vivo approaches, testing with rodent models to determine the functionality of SI 1/28 in assays of inflammatory pain and chronic neuropathic pain [14,15,26]. Based on the structural similarity to the parent compound and reported S1R antagonist SI 1/13 [22], we hypothesized that SI 1/28 would likewise demonstrate anti-inflammatory, anti-allodynic, and anti-hyperalgesic effects in a battery of mouse models of nociception and pain [15,16,26] with reduced liabilities associated with opioids such as drug seeking, respiratory depression, and locomotor impairment or sedation [26,27,28].

## 2. Results

### 2.1. SI 1/28 Induces Dose-Dependent Antinociception in Mouse Models of Visceral Chemical and Inflammatory Nociception

**Figure 1:**
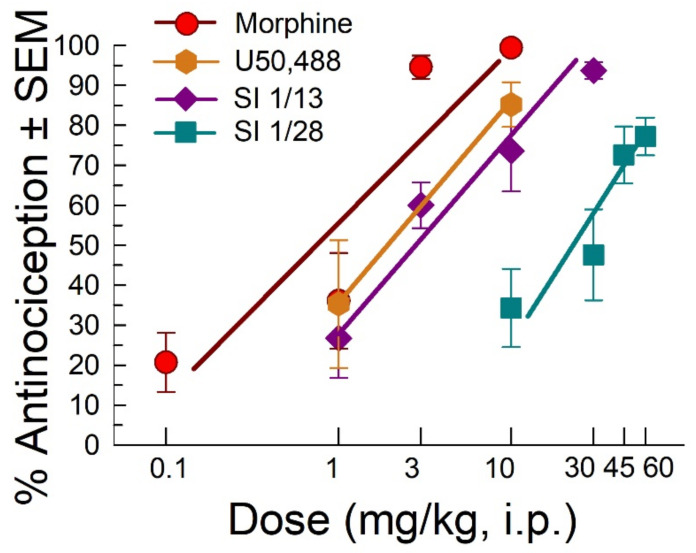
Dose-dependent antinociception of SI 1/28 following i.p. administration in the mouse acetic-acid writhing assay. Opioid agonists morphine, U50,488, and the parent S1R antagonist, SI 1/13 are shown as positive controls. All points represent average response ± SEM at peak effect, 30 min after administration in 7–10 mice.

Following administration through the intraperitoneal (i.p.) route, SI 1/28 dose dependently increased antinociception in the acetic acid writhing test with an ED_50_ (and 95% C.I) value of 27.4 (16.0–43.7) mg/kg, i.p. (Figure 1). These effects were significantly less potent (F_(2, 107)_ = 21.18, *p* < 0.0001; nonlinear regression modeling) than the effects of the parent compound SI 1/13 (ED_50_ (and 95% C.I.) value of 2.67 (1.34–4.27) mg/kg, i.p; Figure 1), the nonselective opioid positive control, morphine (ED_50_ (and 95% C.I.) value of 0.74 (0.40–1.21) mg/kg, i.p; Figure 1) and known kappa opioid receptor (KOR) agonist, U50,488, with an ED_50_ (and 95% C.I.) value of 2.22 (0.63–5.08) mg/kg, i.p. (Figure 1).

**Figure 2:**
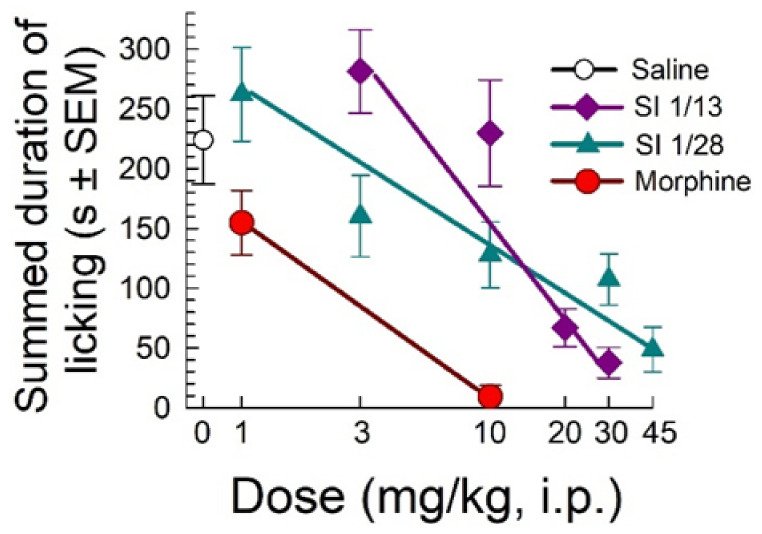
Dose-dependent antinociception of SI 1/28 following i.p. administration in the mouse formalin assay. Control mice were treated with saline (0.9%), morphine or the parent S1R antagonist, SI 1/13. All points represent summed time spent licking ± SEM of 7–13 mice. Note that SI 1/13 data is as previously published in reference [22].

When evaluated in the formalin assay, SI 1/28 demonstrated a dose-dependent decrease in summed duration of licking with an ED_50_ (and 95% C.I.) value of 13.2 (7.42–28.3) mg/kg, i.p (Figure 2). The effects against formalin induced inflammatory pain were significantly less potent (F_(1, 98)_ = 37.8, *p* < 0.0001; nonlinear regression modeling) than demonstrated by control compound morphine (ED_50_ (and 95% C.I.) value = 1.63 (0.85–2.58) mg/kg, i.p; Figure 2), but equivalent to the efficacy of the parent S1R antagonist, SI 1/13 (ED_50_ (and 95% C.I.) value of 12.7 (9.89–16.6) mg/kg, i.p. [22].

### 2.2. Anti-Allodynic Effects of SI 1/28

**Figure 3:**
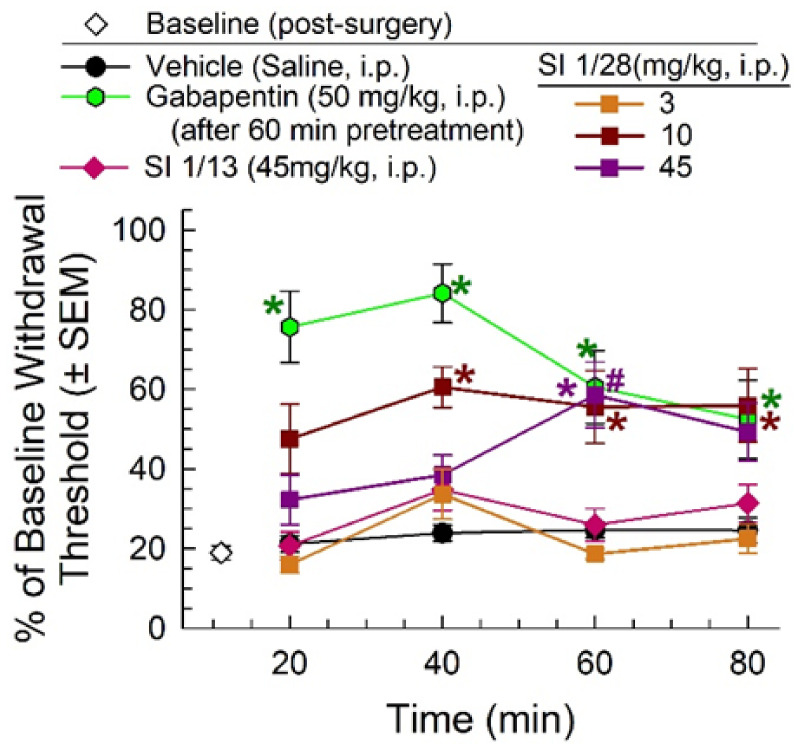
Dose- and time-dependent anti-allodynic activity of SI 1/28 (squares) in the mouse CCI assay of neuropathic pain. Mechanical allodynia produced from sciatic nerve ligation was reduced from 40–80 min (10 mg/kg, i.p., red squares) and 60–80 min (45 mg/kg, i.p.; purple squares), similar to the effect of the positive control, gabapentin (green hexagon). SI 1/28 (45 mg/kg, i.p.; purple squares) was significantly increased compared to the parent compound, SI 1/13 (pink diamonds). *n* = 8–17 for all groups. * = significantly different from vehicle controls; # = significantly different from SI 1/13; *p* < 0.05; two-way RM ANOVA.

One week after chronic constriction nerve injury (CCI), mice displayed characteristic mechanical allodynia (white diamond) that was unchanged by treatment with vehicle, but significantly ameliorated for up to 60 min after a 1 h pretreatment with gabapentin (50 mg/kg, i.p.; time x treatment, F_(3, 66)_ = 7.31, *p* = 0.0003; two-way RM ANOVA with Tukey’s multiple comparisons post hoc test; Figure 3) for up to 60 min. Treatment with SI 1/28 significantly increased paw withdrawal threshold in mice exposed to CCI in a time- (F_(2.49, 119.6)_ = 4.20, *p* = 0.01; two-way RM ANOVA with Tukey’s multiple comparisons post hoc test) and dose-dependent manner (F_(4, 48)_ = 10.2, *p* < 0.0001; two-way RM ANOVA with Tukey’s post hoc test; Figure 3) compared to the saline control. While SI 1/28 did not significantly attenuate allodynia after treatment with a 3 mg/kg, i.p. (orange squares, Figure 3), a 10 mg/kg, i.p. dose was approximately 50–60% effective at reversing mechanical allodynia beginning at 40 min post-administration, and a 45 mg/kg, i.p. dose significantly reduced allodynia at 60 min (*p* = 0.006) and 80 min (*p* = 0.02). Notably, a direct comparison of SI 1/28 and the parent compound SI 1/13 (at doses of 45 mg/kg, i.p.) demonstrated that SI 1/28 was significantly more efficacious in ameliorating mechanical allodynia (F_(3, 81)_ = 3.16, *p* = 0.03; two-way RM ANOVA; Figure 3).

### 2.3. Evaluation of SI 1/28 for Potential Clinical Liabilities

**Figure 4:**
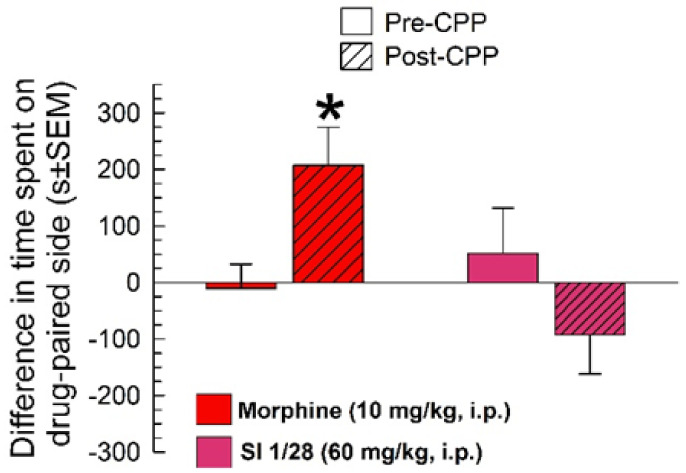
SI 1/28 (60 mg/kg/d, i.p.; *n* = 20) did not demonstrate place conditioning preference or aversion, whereas morphine (10 mg/kg, i.p.) showed significant conditioned place preference (*n* = 22) in the mouse conditioned place preference assay. * = post-conditioning response (striped bars) significantly different from matching pre-CPP response (matching open bars), *p* = 0.003; two-way RM ANOVA w/Sidak’s post hoc test.

In the conditioned place preference (CPP) assay, morphine (10 mg/kg, i.p.) produced significant differences between pre- and post-conditioning responses (conditioning x treatment: F_(1, 40)_ = 15.3, *p* = 0.0003; two-way RM ANOVA with Sidak’s multiple comparisons post hoc test; Figure 4). In contrast, SI 1/28 did not demonstrate significant conditioned place preference (*p* = 0.07).

**Figure 5:**
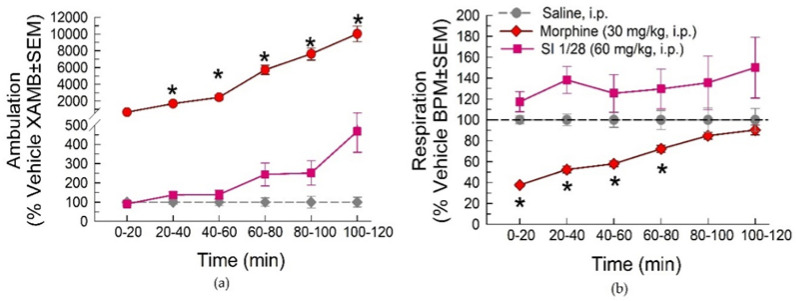
Dose- and time-dependent (**a**) spontaneous ambulation and (**b**) respiratory effects of morphine (30 mg/kg, i.p.; red circles) or SI 1/28 (60 mg/kg, i.p.; pink squares) evaluated in the CLAMS/Oxymax system with C57BL6/J mice. * = significantly greater than vehicle effect (gray dashed line), *p* < 0.05; two-way ANOVA w/Dunnett’s post hoc test. *n* = 12 mice/group. Data presentenced as % vehicle response ± SEM; ambulation, XAMB (**a**) or breaths per minute, BPM (**b**).

In evaluation of spontaneous locomotor activity and respiratory effects using the Comprehensive Lab Animal Monitoring System (CLAMS), morphine (30 mg/kg, i.p.) demonstrated significant increases in ambulation from 20–120 min post-treatment (treatment: F_(2, 33)_ = 103.3; *p* < 0.0001 and time: F_(5, 165)_ = 114.7; *p* < 0.0001; two-way RM ANOVA with Dunnett’s multiple comparisons post hoc test; Figure 5a). In contrast, SI 1/28 (60 mg/kg, i.p.) produced no significant changes in spontaneous ambulation at any time point (*p* > 0.05). Morphine also produced significant reductions in respiration rate between 0–80 min post-injection (treatment: F_(2, 33)_ = 9.25; *p* < 0.0006 and time: F_(5, 165)_ = 5.37; *p* < 0.0001; two-way ANOVA with Dunnett’s post hoc test; Figure 5b). Again, SI 1/28 had no significant effects on breaths per minute compared to the saline control (*p* > 0.05) for all time points.

**Figure 6:**
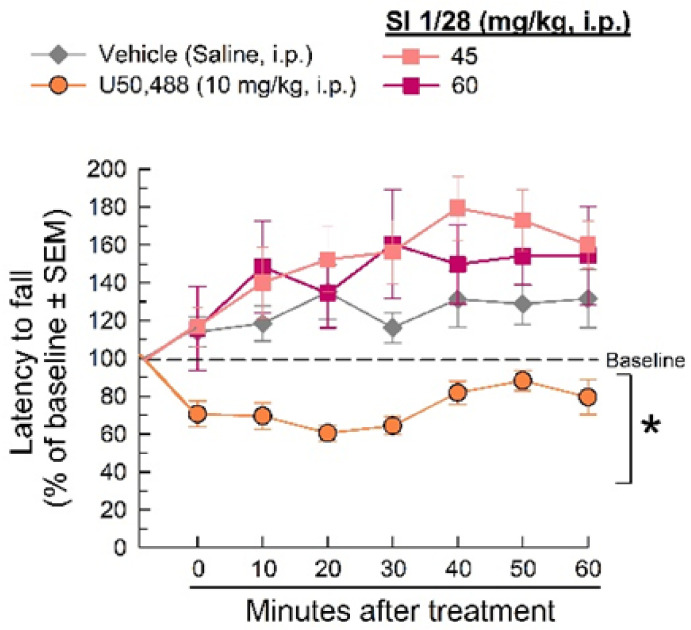
Dose- and time-dependent effects of SI 1/28 (squares) after 45 and 60 mg/kg i.p. administration in the mouse rotarod assay. U50,488 (10 mg/kg, i.p; orange circles.) is the positive control. * = significantly different than vehicle (0.9% saline; gray diamonds), *p* < 0.05; two-way ANOVA w/Dunnett’s post hoc test; *n* = 8–12 mice/treatment.

Changes in evoked or sedative-like effects were evaluated using the rotarod apparatus. The KOR agonist, U50,488, impaired locomotion compared to saline (treatment: F_(3, 259)_ = 34.7, *p* < 0.0001 and time: F_(6, 259)_ = 3.04, *p* = 0.0068, Two-way RM ANOVA with Dunnett’s post hoc test; Figure 6). However, neither the 45 or 60 mg/kg (i.p.) doses of SI 1/28 impaired evoked locomotion or produced sedative-like effects as compared to the vehicle control (*p* > 0.05).

## 3. Discussion

The persistence of the opioid epidemic continues to drive an enormous need to produce nonopioid therapeutics for pain [29,30]. Preclinical and clinical research with S1R antagonists has suggested their considerable promise for the potential treatment of chronic neuropathic pain [13,15,16,26,29,31].

Consistent with previous studies conducted with S1R antagonists, SI 1/28 attenuated acute non-reflexive inflammatory pain in the formalin and acetic acid writhing assays [26,32]. After an initial, brief acute pain response associated with the administration of formalin in the test (phase I, thought to be caused by immediate stimulation of peripheral C fibers) [33], phase II is characterized as an extended tonic period of nociception [34,35] that is associated with neuronal sensitization in the spinal cord attributed to stimulation of the afferent nociceptors [36]. The blockade of formalin-induced pain in phase II by SI 1/28 administration is consistent with the reported involvement of S1R in sensitization to nociception [14,37]. In contrast, visceral pain associated with acetic acid administration in the writhing test is attributed to direct activation of the nociceptors in the colon [38]. The hyperalgesia produced by intraperitoneal (i.p.) administered acetic acid in wild-type mice was dose dependently reversed by SI 1/28 pretreatment. Together, these current data suggest that the function of SI 1/28 is in line with antinociceptive properties displayed by other S1R antagonists [15,17,26,39]. 

While moderately effective, SI 1/28 proved more efficacious than SI 1/13 at alleviating neuropathic pain in the CCI assay. A common clinical therapeutic for neuropathic pain, gabapentin (Neurontin), exhibited strong anti-allodynic effects, but it must be noted this was measured after a 1 h pretreatment to avoid known sedative effects [3]. SI 1/28 failed to produce anti-allodynic activity at a low dose of 3 mg/kg, i.p., which still demonstrated antinociceptive efficacy in the formalin and acetic acid writhing assays. Failure to produce consistent results across the nociceptive assays points to limitations inherent to fully describing the method of action associated with SI 1/28. In the case of G-protein coupled receptors (GPCRs), these type of effects might be interpreted as due to effects of a partial agonist, many of which have been shown to be effective in the treatment of acute and chronic pain [40]. However, S1Rs are currently classified as ligand-operated chaperone receptors and not GPCRs, and are thus thought to mediate analgesia through interactions with a host of proteins influencing nociception [13,14]. Understandably, this complicates a ready understanding of the pharmacology and precise mechanisms by which S1R ligands produce antinociception and anti-allodynic effects. As new and more selective antagonists such as SI 1/28 become available, and given the therapeutic promise of existing S1R antagonists such as MR309 [13], further evaluation of the pharmacological mechanisms by which S1R antagonists mediate analgesia are warranted. 

Due to their status as chaperone receptors, sigma receptors are not bound by established mechanistic pharmacological rules governing receptor function, raising questions as to the precise mechanisms underlying the action of S1R antagonists. Historically, antinociceptive effects of S1R antagonists have been attributed the S1R’s ability to interact with other proteins such as NMDA [41] and opioid [8] receptors, and ion channels such as calcium and potassium channels [42]. Interaction with other proteins could account for some of the antagonistic effects in inflammatory assays, while attenuating anti-neuropathic effects. Elucidating the affinity for other receptor and ion channel targets associated with antinociception is beyond the scope of this study but remains a priority for further investigation. 

“Gold standards” for analgesia such as morphine and gabapentin produce some therapeutic benefit against chronic pain, but are associated with liabilities such as decreased respiration, locomotor, and in some cases addiction and death [6,43]. SI 1/28 was assessed for adverse effects such as reward (as conditioned place preference), respiratory depression (as breath rate), and locomotor impairment. In line with previous studies, when tested in the Comprehensive Lab Animal Monitoring System, morphine demonstrated expected spontaneous hyperlocomotion and severely decreased respiration [44,45]. In contrast, SI 1/28 produced no adverse respiratory effects or increased locomotor activity. The current results contrast with previous observations with the selective S1R antagonist CM-304, which displayed hyperventilation at low doses and hypoventilation after treatment with high doses [26]. Cirino and co-authors [26] speculated that S1R concentrations in brain regions that control sedation/arousal (i.e., hypothalamus and medulla) [46] may account for these effects; however, the correlation between the S1R and respiration is not fully understood and warrants further investigation. Moreover, CM-304 retained modest affinity for the S2R (with a *K*_i_ value of 364 nM, [18]) not possessed by SI 1/28 which could contribute to the discrepancy. Regardless, to confirm the lack of locomotor activity, SI 1/28 was further tested for its ability to impair induced locomotor activity in the rotarod assay. In this case, the S1R antagonist exhibited similar effects to CM-304 with no disruption in evoked locomotor activity [26]. 

Given reports that rodents self-administer S1R agonists such as PRE-084 at higher doses [47], potential rewarding properties of SI 1/28 were assessed at a supratherapeutic dose, 60 mg/kg. While self-administration studies of SI 1/28 were beyond the scope of the current study, potential rewarding effects were evaluated in the CPP assay. Unlike morphine, which displayed typical rewarding effects in CPP [48,49], SI 1/28 did not produce conditioned place preference. These effects were in accordance with literature demonstrating that S1R antagonists produce neither conditioned place preference nor conditioned place aversion [26]. While the exact mechanism of S1R antagonist activity requires further investigation, an interaction with reward circuitry has been inferred from reports where S1R antagonist treatment blocks sensitization to the locomotor effects of methamphetamine [50], cocaine-seeking behavior and neurotoxicity [51]. Although beyond the scope of the current characterization, further investigation is warranted to evaluate if SI 1/28 modulates the reinforcing effects of methamphetamine and cocaine as reported in the presence of other S1R antagonists.

Currently, these data support the development of S1R-selective antagonists as therapeutics for the treatment of chronic pain. Justifying this, S1R is highly expressed in key areas in the central and peripheral nervous systems regulating the transduction, conduction and perception of pain signals, notably in the dorsal root ganglia [12,31]. Precise mechanistic insights into how S1R modulates neuropathic and acute inflammatory nociception have yet to be elucidated. Of interest, S1R antagonists have proven less effective at modifying normal sensitivity thresholds to nociception, perhaps consistent with the reduced liabilities observed such as sedation. 

## 4. Materials and Methods

### 4.1. Subjects

Male C57BL/6J and CD-1 with ages ranging 8–12 weeks were housed five to a cage were used. C57BL/6J mice were used in the locomotor and respiration [52], rotarod [53], acetic acid writhing [26], and conditioned place preference [54,55] assays. CD-1 mice were used to verify antinociception in the formalin and chronic constriction nerve injury assay of neuropathic pain [56,57,58,59]. 

ARRIVE guidelines were used to execute and report all animal studies [60]. Treatment groups were blinded and randomly assigned animals. Animals were housed on a 12:12-h light/dark cycle (lights off at 7:00 pm) with *ad libitum* access to food and water except during experimental sessions. All procedures were Institutional Animal Care and Use Committee (University of Florida) preapproved and conducted in according to the 2011 NIH Guide for the Care and Use of Laboratory Animals.

### 4.2. Materials, Drug Preparation, and Administration 

SI 1/28 was resynthesized as a free base following our previous reported synthetic procedures [22,61,62], and then converted into oxalate salt for in vivo testing. The synthetic procedure and experimental data of 1-(4-{[4-(hydroxymethyl)phenyl]methyl}piperazin-1-yl)-5-phenylpentan-1-one oxalate are reported in the Supplementary Materials. All other chemicals and drugs were purchased from Sigma-Aldrich (St. Louis, MO, USA). Sterile saline (0.9%) was used to dissolve morphine and known kappa opioid receptor (KOR) agonist U50488. Gabapentin and SI 1/28 were dissolved in 5% dimethyl sulfoxide (DMSO)/95% saline. All drugs were administered intraperitoneally (i.p.) in a volume of 0.25 mL per 25 g body weight. 

### 4.3. Behavioral Assays

#### 4.3.1. Acetic Acid Stretching Assay

Chemically induced visceral pain was assessed in C57BL/6J mice using the acetic acid stretching assay as previously described [53,63]. SI 1/28 (10–60 mg/kg), U50,488 (1 and 10 mg/kg), or morphine (0.1–10 mg/kg) was administered 25 min. prior to i.p. administration of 0.9% acetic acid (0.25 mL per 25 g body wt.) to each mouse. After 5 min, the number of stretches presented in each mouse was counted for 15 min. Antinociception was calculated by the formula:

% antinociception = ([{average stretches in the vehicle group} − {number of stretches in each test mouse}]/[average stretches in vehicle group]) × 100.

#### 4.3.2. Formalin Assay

Inflammatory antinociception was evaluated with the use of the formalin assay in C57BL/6J mice as previously described [64]. SI 1/28 (1–45 mg/kg), SI 1/13 (3–30 mg/kg), morphine (1 and 10 mg/kg), or saline (0.9%) were administered as a 10 min. pretreatment prior to an intraplantar (i.pl.) injection of 5% formalin (2.5 μg in 15 μL) administered into the right hind paw. Time spent licking the right hind paw was recorded in 5 min intervals for 60 min following injection. The last 55 min of assessment was used to determine inflammatory response stimulus. Data were analyzed as area under the curve (AUC), representing summed time mice spent licking the right hind paw.

#### 4.3.3. Chronic Constriction Injury (CCI)

CD-1 mice were used in the CCI assay. Mice were anesthetized with isoflurane as previously described [26,65]. Mice then underwent surgery where a small skin incision was made along the exterior of the biceps femoris of the right hind paw [65]. The muscle was split using blunt forceps and the right sciatic nerve was exposed. Two opposite facing 0.1–10 µL pipette tips were placed under the sciatic nerve to allow for the ease of passing of two sutures under the nerve, 1 mm apart. The ligatures were tied loosely around the nerve and secured with two knots. The skin incision was closed with two 9 mm skin staples. After a 7-day recovery period, the mice underwent baseline von Frey testing to confirm acquisition of hyperalgesia or mechanical allodynia. 

Von Frey testing was performed as previously described [64,66,67,68]. Baseline mechanical allodynia readings were taken using the application of filaments with increasing pressure (0.4–6 g) to the plantar of the hind paw of mice prior to drug administration. Lower withdrawal thresholds of allodynia were considered neuropathic. Vehicle control (saline), gabapentin (50 mg/kg), or SI 1/28 (3–45 mg/kg) were administered (i.p.), and paw-withdrawal thresholds were recorded from in 20 min increments until 80 min post-injection in both the contralateral and ipsilateral hind paws. Time points were measured in triplicate with licking, shaking, or paw withdrawal as indication of a response. It should be noted that gabapentin was administered as a 60 min pretreatment prior to testing in the CCI assay to avoid confounding sedative effects [69]. 

#### 4.3.4. Conditioned Place Preference (CPP)

Automated, three-compartment place preference chambers were used to evaluate CPP in C57BL/6J mice. A 2day counterbalanced conditioning design [70] was employed with initial place preference evaluation being tested 24 h prior to place conditioning. In initial preference testing, the mice were allowed free access to all compartments of the apparatus for 30 min and total time spent in each compartment was recorded. For 2 days following initial preference evaluation, the mice were administered vehicle (5% DMSO and 95% saline) then confined into an outer compartment of the apparatus for 40 min. Four h after saline administration, the mice were administered (i.p.) either morphine (10 mg/kg) or SI 1/28 (60 mg/kg) then confined in the opposite outer compartment for 40 min. Conditioning restrictions were repeated precisely on day 2 of place conditioning. 24 h after the second day of conditioning, final place preference was evaluated. Mice were allowed to roam freely between all chambers for 30 min. Data are expressed as the difference in time spent in the drug-paired and vehicle-paired compartments. Positive values represent conditioned preference, whereas negative values are considered conditioned aversion for the drug-paired side. 

#### 4.3.5. Respiratory Depression and Spontaneous Locomotor Testing with CLAMS

Respiration rate and spontaneous locomotor activity were calculated by the computer automated, Comprehensive Lab Animal Monitoring System (CLAMS; Columbus Instruments, Columbus, OH, USA,) as described previously [52]. Unrestrained mice were allowed to habituate individually in sealed cages connected to the system for 60 min preceding testing for initial mouse readings. Mice were then administered i.p. SI 1/28 (60 mg/kg), morphine (30 mg/kg), or saline (0.9%), and placed back into the CLAMS testing units for 120 min. A built-in pressure transducer within the seal cages was used to measure respiration rates (breaths/min). Spontaneous locomotion was measure via infrared photobeams located at the bottom of each cage. Ambulation was counted as the number of photobeam breaks. All data are expressed as percent of vehicle control response.

#### 4.3.6. Rotarod Assay to Assess Motor Coordination

Impairment of evoked locomotor activity or potential sedative effects were measured in the rotarod coordination assay as previously described [53]. Seven training trials were performed where the last training trial was used as a baseline of performance. Vehicle, U50,488 (10 mg/kg), or SI 1/28 (60 mg/kg) was administered (i.p.) then assessed in 10 min in accelerated speed trials (180 s max latency at 0−20 rpm) for 60 min. Latency to fall was measured in seconds. Data are reported as the average % difference from each mouse’s baseline latency reading. Reduced latencies to fall in the rotarod test suggest impaired motor coordination or sedation.

#### 4.3.7. Statistical Analysis 

Data are presented as mean ± SEM. GraphPad Prism 7.0 software was used for statistical analysis. Data were analyzed using either one-way or two-way ANOVA with the appropriate post hoc test (Dunnett’s, Sidak’s, or Tukey’s) where *p* < 0.05 was considered significant. Linear regression was used to determine ED_50_ values, and 95% confidence intervals of dose response cures presented in formalin and acetic acid writhing assays. Condition place preference data are represented as the difference between time spent in the drug pair compartment and the vehicle paired compartment between pre- and post-conditioning. CLAMS data are reported as a % of matching vehicle control responses. The rotarod data are expressed as the % change from baseline performance for each animal’s baseline response. 

## 5. Conclusions

The S1R antagonist, SI 1/28, proved efficacious in the treatment of acute inflammatory and visceral nociception and chronic neuropathy, while displaying no significant liabilities of reward, sedation or respiratory depression at therapeutic doses.

## Data Availability

Datasets generated for this study are available on request to the corresponding authors.

## Supplementary Materials

The following are available online at https://www.mdpi.com/article/10.3390/ijms23020615/s1, synthetic procedure for the preparation of 1-(4-{[4-(hydroxymethyl)phenyl]methyl}piperazin-1-yl)-5-phenylpentan-1-one oxalate (SI 1/28), ^1^H NMR and APT spectra of SI 1/28.
