# Supplementary material for: Examination of the Novel Sigma-1 Receptor Antagonist, SI 1/28, for Antinociceptive and Anti-allodynic Efficacy against Multiple Types of Nociception with Fewer Liabilities of Use
Source: Int J Mol Sci. 2022 Jan 6;23(2):615. doi: 10.3390/ijms23020615 (PMC8775934; doi:10.3390/ijms23020615)
Supplement: Supplementary file 1 [file ijms-23-00615-s001.zip › ijms-1530683-supplementary.pdf]

International Journal of
*Molecular Sciences*

MDPI

*Supplementary Materials*

# Examination of the Novel Sigma-1 Receptor Antagonist, SI 1/28, for Antinociceptive and Anti-allodynic Efficacy against Multiple Types of Nociception with Fewer Liabilities of Use

**Lisa L. Wilson [1], Shainnel O. Eans [1], Insitar Ramadan-Siraj [1], Maria N. Modica [2], Giuseppe Romeo [2], Sebastiano Intagliata [2]* and Jay P. McLaughlin [1]***

[1] Department of Pharmacodynamics, The University of Florida, Gainesville, FL 32610, USA; lisawilson@ufl.edu (L.L.W); shaieans@cop.ufl.edu (S.O.E); I.SirajRamadan@hotmail.com (I..R.S.); jmclaughlin@cop.ufl.edu (J.P.M)

[2] Department of Drug and Health Sciences, University of Catania, 95125 Catania, Italy; mmodica@unict.it (M.N.M); gromeo@unict.it (G.R.); s.intagliata@unict.it (S.I.)

* Correspondence: s.intagliata@unict.it; Tel.: +39-095-738-4053 (S.I.); jmclaughlin@cop.ufl.edu; +1-352-273-7207 (J.P.M)

**Table of contents**


| | |
|---|---|
| Synthetic procedure for the preparation of 1-(4-{[4-(hydroxymethyl)phenyl]methyl}piperazin-1-yl)-5-phenylpentan-1-one oxalate (**SI 1/28**) | S2 |
| Figures S1, S2: $^1$H NMR and APT spectra of **SI 1/28** | S3 |
| Reference | S4 |

## Chemistry

All chemicals and solvents were reagent grade and were purchased from commercial vendors. Melting point was determined in an IA9200 Electrothermal apparatus equipped with a digital thermometer in glass capillary tubes and is uncorrected. The infrared (IR) spectrum was recorded in KBr disk on a Perkin Elmer 1600 Series FT-IR spectrometer. $^1$H NMR and $^{13}$C NMR spectra were recorded on a Varian Inova Unity 500 spectrometer, using a methanol-$d_4$ solution. Chemical shifts are given in δ values (ppm), using tetramethylsilane as the internal standard; coupling constants (*J*) are given in hertz (Hz). Signal multiplicities are characterized as s (singlet), t (triplet), m (multiplet), or br (broad signal). Elemental analysis for C, H, and N was within ± 0.4% of theoretical value and was performed on a Carlo Erba Elemental Analyzer Mod. 1108 apparatus.

*Synthesis of 1-(4-{[4-(hydroxymethyl)phenyl]methyl}piperazin-1-yl)-5-phenylpentan-1-one oxalate (**SI 1/28**).* 1-(4-{[4-(hydroxymethyl)phenyl]methyl}piperazin-1-yl)-5-phenylpentan-1-one free base was reprepared in a bulk amount following a previously reported procedure [1]. Subsequently, a solution of oxalic acid (76.0 mg, 0.84 mmol) in methanol (1 mL) was poured into a stirred solution of the benzylpiperazine derivative (308.9 mg, 0.84 mmol) in methanol (1 mL). The mixed solution was stirred for 3 h, at room temperature, until the solution gradually became cloudy. Diethyl ether (15 mL) was then added, resulting in a white precipitate which was left to stand for 1 h to allow it to settle. The solid was collected by filtration under reduced pressure, washed twice with fresh diethyl ether, and dried to remove any residual traces of solvents to afford **SI 1/28** (238.6 mg, 62%) as a white pure solid. Mp: 144.7–145.9 °C; IR (KBr, selected lines) cm$^{-1}$: 3412, 2977, 1716, 1695, 1643, 1458, 1417, 1272, 1039, 720, 694. $^1$H NMR (500 MHz, $CD_3OD$): δ 7.45 (br, 4H), 7.27 – 7.10 (m, 5H), 4.63 (s, 2H, $CH_2$OH), 4.26 (s, 2H), 3.91 – 3.63 (m, 4H), 3.25 – 3.07 (m, 4H), 2.63 (t, *J* = 7.1 Hz, 2H), 2.42 (t, *J* = 7.1 Hz, 2H), 1.73 – 1.54 (m, 4H). $^{13}$C NMR (126 MHz, $CD_3OD$): δ 174.0, 166.2, 145.0, 143.5, 132.3, 129.5, 129.4, 129.3, 128.5, 126.8, 64.5, 61.5, 52.6, 52.5, 43.8, 39.8, 36.5, 33.5, 32.1, 25.7. Anal. Calcd. for $C_{23}H_{30}N_2O_2 \cdot C_2H_2O_4$: C, 65.77; H, 7.07; N, 6.14. Found: C, 65.58; H, 7.10; N, 6.13.

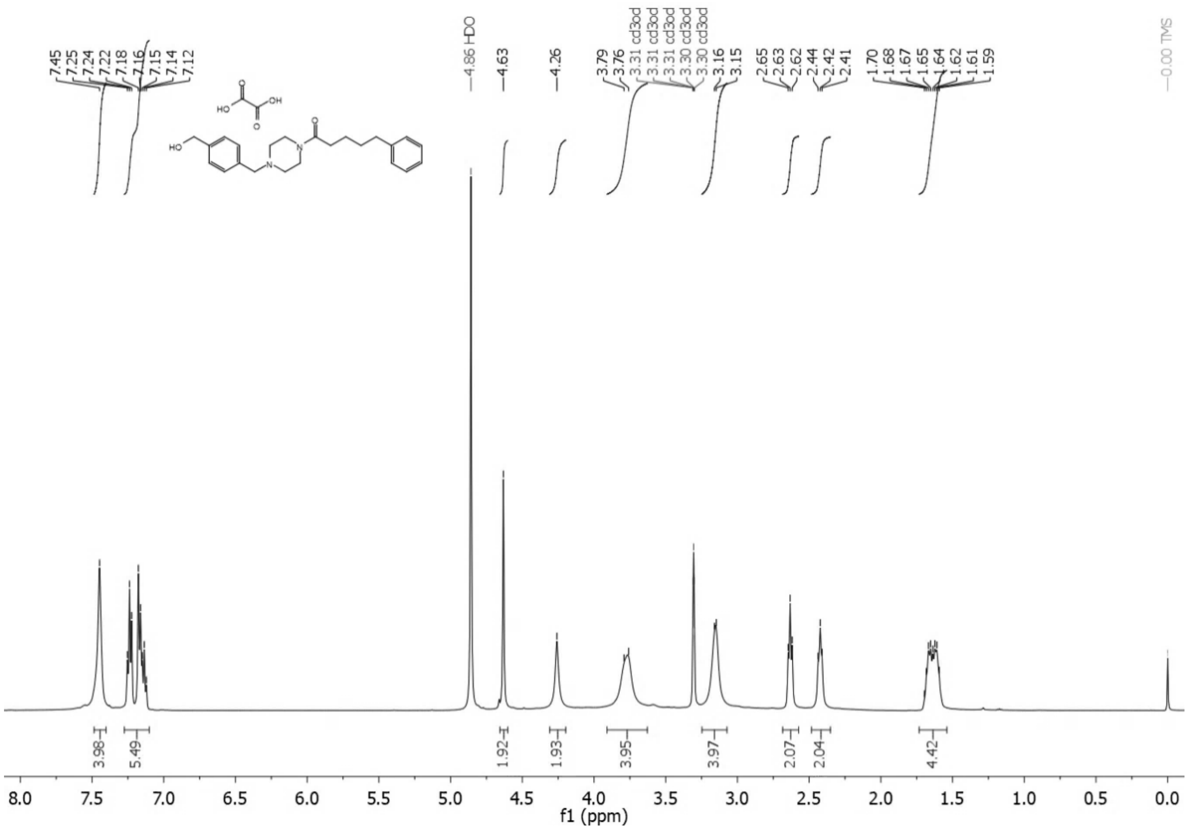


**Figure S1.** $^{1}$H NMR spectrum of **SI 1/28**

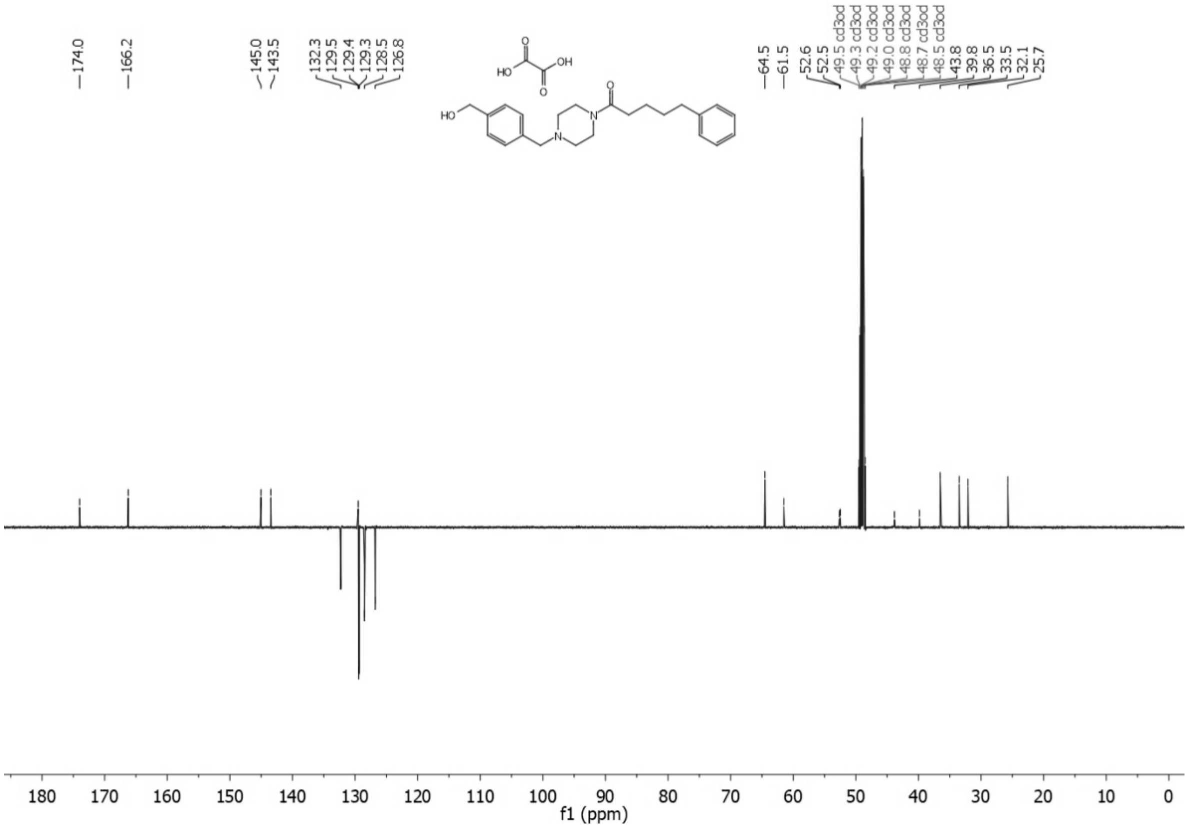


**Figure S2.** APT spectrum of **SI 1/28**

## Reference:


1. Romeo, G.; Bonanno, F.; Wilson, L. L.; Arena, E.; Modica, M. N.; Pittalà, V.; Salerno, L.; Prezzavento, O.; McLaughlin, J. P.; Intagliata, S., Development of New Benzylpiperazine Derivatives as σ(1) Receptor Ligands with in Vivo Antinociceptive and Anti-Allodynic Effects. *ACS chemical neuroscience* **2021,** 12, (11), 2003-2012.
